# Mycofier: a new machine learning-based classifier for fungal ITS sequences

**DOI:** 10.1186/s13104-016-2203-3

**Published:** 2016-08-11

**Authors:** Luisa Delgado-Serrano, Silvia Restrepo, Jose Ricardo Bustos, Maria Mercedes Zambrano, Juan Manuel Anzola

**Affiliations:** 1 Bioinformatics & Computational Biology, Corporación CorpoGen, Bogotá, DC Colombia; 2 Department of Biological Sciences, Universidad de Los Andes, Bogotá, DC Colombia

**Keywords:** Fungal ITS1, Fungi, Naive Bayes classifier, Fungal diversity

## Abstract

**Background:**

The taxonomic and phylogenetic classification based on sequence analysis of the ITS1 genomic region has become a crucial component of fungal ecology and diversity studies. Nowadays, there is no accurate alignment-free classification tool for fungal ITS1 sequences for large environmental surveys. This study describes the development of a machine learning-based classifier for the taxonomical assignment of fungal ITS1 sequences at the genus level.

**Results:**

A fungal ITS1 sequence database was built using curated data. Training and test sets were generated from it. A Naïve Bayesian classifier was built using features from the primary sequence with an accuracy of 87 % in the classification at the genus level.

**Conclusions:**

The final model was based on a Naïve Bayes algorithm using ITS1 sequences from 510 fungal genera. This classifier, denoted as Mycofier, provides similar classification accuracy compared to BLASTN, but the database used for the classification contains curated data and the tool, independent of alignment, is more efficient and contributes to the field, given the lack of an accurate classification tool for large data from fungal ITS1 sequences. The software and source code for Mycofier are freely available at https://github.com/ldelgado-serrano/mycofier.git.

**Electronic supplementary material:**

The online version of this article (doi:10.1186/s13104-016-2203-3) contains supplementary material, which is available to authorized users.

## Background

Fungi represent an essential functional component of Earth’s biodiversity, not only because of their roles as decomposers, mutualists and pathogens, but also because they are the second most speciose eukaryotic kingdom [1, 2]. Several rRNA genes have been used to explore their diversity and used as method for their identification; these include the small ribosomal subunit (SSU), the large ribosomal subunit (LSU) and the internal transcribed spacer (ITS) [3]. The ITS region has a higher PCR amplification success rate compared with other phylogenetic markers such as RPB1, SSU and LSU; also, it has a species discrimination power throughout the entire fungal kingdom and a defined barcode gap. Given these advantages, Schoch et al. [4] proposed ITS as the standard barcode for fungi. ITS includes the ITS1 and ITS2 regions, separated by the 5.8S gene in the nuclear rDNA repeat unit [5]. The entire ITS region has commonly been sequenced with traditional Sanger approaches with a typical amplicon that ranges between 450 and 700 bp. Either the ITS1 or the ITS2 regions have been targeted in recent high-throughput sequencing studies because the entire ITS region is still too long for illumina sequencing, the predominant method today [6–8]. In particular, the ITS1 region has been used recently for fungal phylogeny, taxonomic placement and for environmental surveys [9–11]. ITS1 is a hypervariable region that allows species identification and subgeneric phylogenetic inference [12–14]. However, pairwise alignments are less effective and show a comparative lower efficiency than alignment free methods when it comes to taxonomic assignment of sequences that show a high sequence divergence between their members such as ITS1.

Machine learning-based algorithms have been used as a response to the problems in computational biology such as classification of biological data. Among these tools, the Naïve Bayesian classification method is simple yet can be extremely efficient. This type of classifier is based on the Bayes theorem and “Naïve” refers to the assumption that data attributes are independent from each other. Even when the independency of data attributes is violated, the Bayesian method can still be optimal [15]. A Naïve Bayes classifier assigns an object to a class based on the probability the object has according to its features. In bioinformatics, the Naïve Bayesian classification method has been reported to perform well on problems similar to the classification of sequence data, such as the Ribosomal Database Project (RDP) Classifier [16].

The aim of this work was to develop a machine learning-based classifier for classifying fungal ITS1 sequences according to the NCBI taxonomy at the genus level. Here we explored the use of Naïve Bayes with different features and parameters in order to develop a classifier for ecological studies using high-throughput data.

## Results

### The ITS1 database and final dataset

**Fig. 1:**
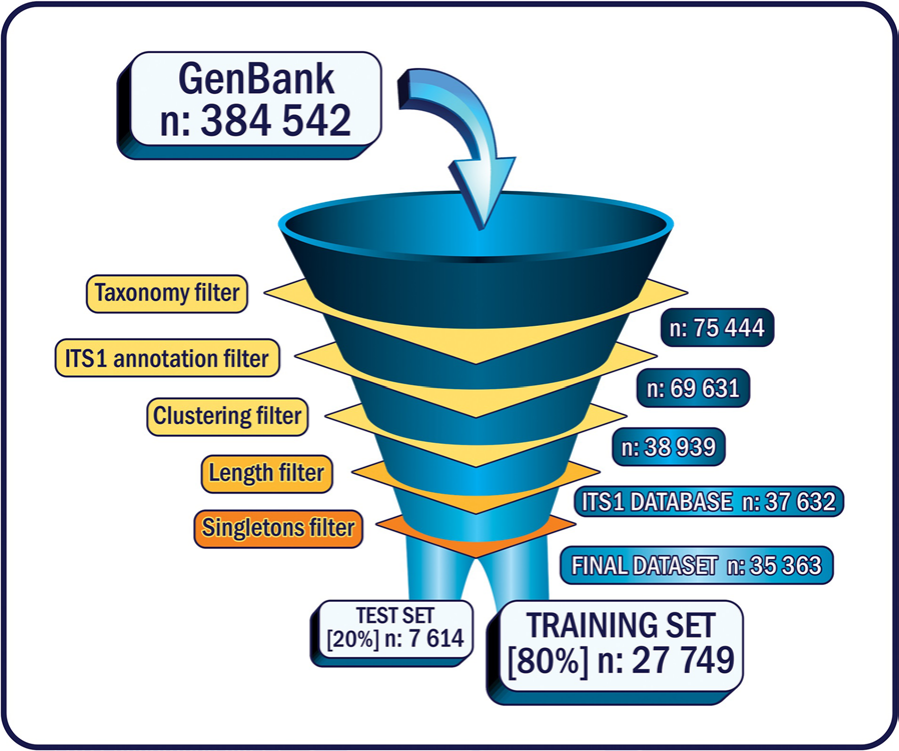
Pipeline scheme for fungal ITS1 database and data set construction

A total of 37,632 fungal ITS sequences were obtained from the manual curation process. Table 1 shows the composition of the entire database, including taxa represented by less than 5 sequences. All of these were later removed from the analysis. The remaining 35,363 sequences were used for the construction of the training and test sets (Fig. 1).

**Table 1:** Taxonomic composition of the fungal ITS1 database

Classification level	No. of taxa
Phylum	7
Class	32
Order	121
Family	383
Genus	2112

In total, there were 37,632 sequences

**Fig. 2:**
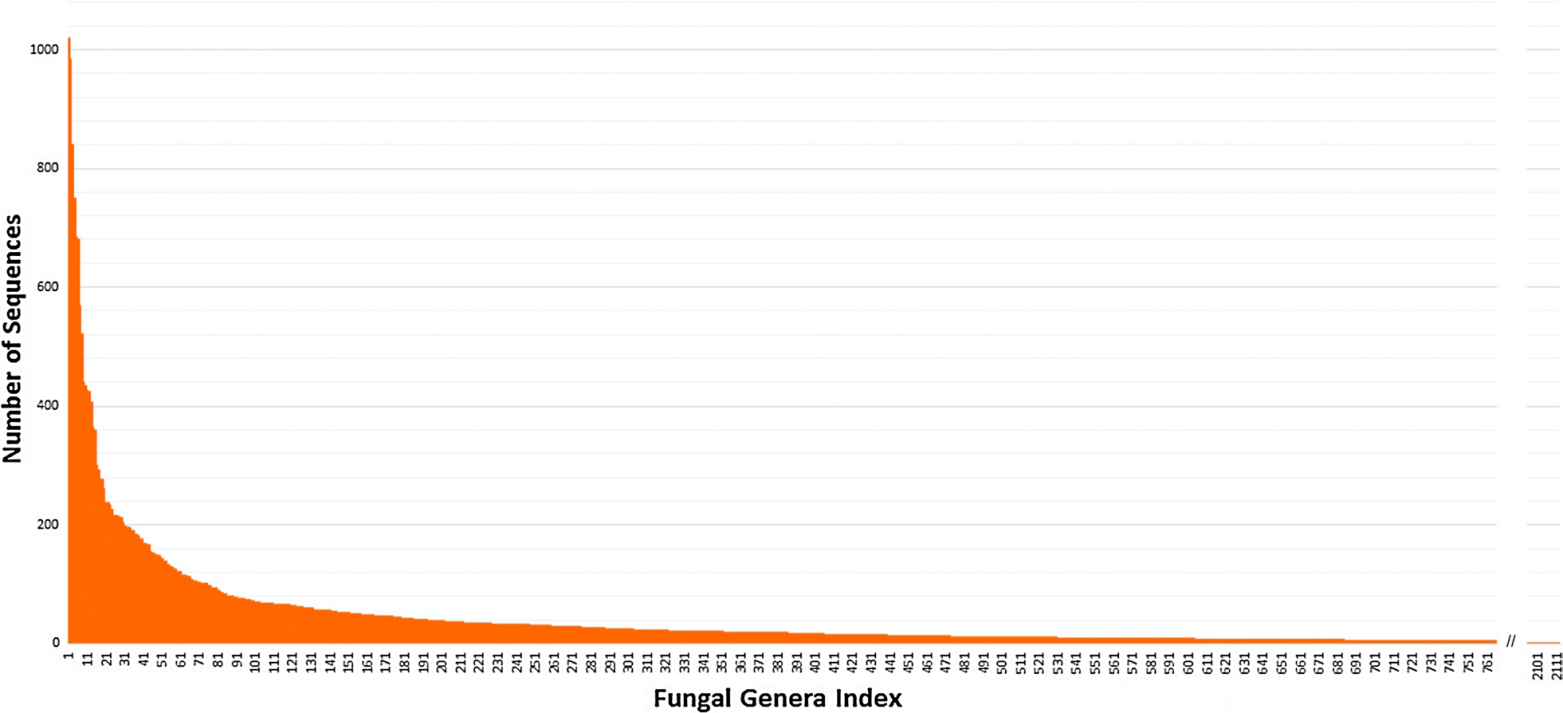
Number of sequences throughout the 2112 fungal genera in the ITS1 database

The ITS1 database was heterogeneous in terms of number of sequences by genera since there were a 25 % of genera with more than 10 sequences and 61 % of genera with <5 sequences (Fig. 2).

The final dataset for classification analyses included sequences from 822 validated fungal genera (about 39 % of the ITS1 database genera) spanning 28 classes (and 3 orders *incertae sedis*) and 6 phyla (and 2 subphyla *incertae sedis*). The taxonomic composition of the entire Weka dataset (training and test sets) is shown in Table 2. Most of the sequences (~67 %) represented 14 classes within the Ascomycota. Twenty-nine percent of the sequences represented 8 classes within the Basidiomycota. Among the most abundant classes of fungi were Agaricomycetes (25.3 %), Sordariomycetes (23.02 %), Lecanoromycetes (12.95 %), Dothideomycetes (11.75 %), and Eurotiomycetes (9.24 %). This result reflects the bias existing towards these groups in databases (Table 2).

**Table 2:** Taxonomic composition of the 35,363-sequence fungal ITS1 region dataset used for the classifier analyses

Phylum (% of sequences)	Class	No. of genera	No. (%) of sequences
Basidiomycota (29.37)	Agaricomycetes	207	8942 (25.3)
	Agaricostilbomycetes	3	30 (0.08)
	Cystobasidiomycetes	2	42 (0,12)
	Exobasidiomycetes	5	151 (0.43)
	Microbotryomycetes	5	184 (0.52)
	Pucciniomycetes	12	527 (1.49)
	Tremellomycetes	12	384 (1.09)
	Ustilaginomycetes	4	65 (0.18)
Ascomycota (67.11)	Arthoniomycetes	4	168 (0.47)
	Coniocybomycetes	1	41 (0.12)
	Dothideomycetes	105	4157 (11.75)
	Eurotiomycetes	59	3267 (9.24)
	Geoglossomycetes	1	7 (0.02)
	Lecanoromycetes	123	4579 (12.95)
	Leotiomycetes	51	1414 (4)
	Orbiliomycetes	7	143 (0.40)
	Pezizomycetes	19	814 (2.30)
	Pneumocystidomycetes	1	13 (0.04)
	Saccharomycetes	29	1013 (2.86)
	Schizosaccharomycetes	1	5 (0.01)
	Sordariomycetes	144	8141 (23.02)
	Taphrinomycetes	1	5 (0.01)
Glomeromycota (1.58)	Archaeosporomycetes	2	52 (0.15)
	Glomeromycetes	6	483 (1.37)
	Paraglomeromycetes	1	17 (0.05)
Blastocladiomycota (0.04)	Blastocladiomycetes	1	15 (0.04)
Chytridiomycota (0.24)	Chytridiomycetes	5	83 (0.23)
Neocallimastigomycota (0.03)	Neocallimastigomycetes	1	11 (0.03)
Entomophthoromycotina (0.08) (Subphylum)	Entomophthorales (Order)	3	29 (0.08)
Mucoromycotina (1.55) (Subphylum)	Mucorales (Order)	15	551 (1.56)
	Endogonales (Order)	1	5 (0.01)

This dataset was split afterwards in order to construct the training set (80 % of the dataset) and test set (20 % of it) by picking a random sequence from the Weka dataset without replacement until the test set was 20 % of the size of the Weka dataset (Fig. 1).

### Classification accuracy

#### Effect of *k*-mer size

We conducted a systematic search of features in order to determine what features would result in the best performing vector. The first feature evaluated was the *k*-mer feature that refers to all the possible subsequences (4 ^*k*^) of length *k* from a sequence. We changed the size of the *k*-mer of the primary sequence, starting from 2 (dimers) up to a value of 6 and then calculated their frequencies. The length of the sequence normalized by the average length and the percent GC content were also used as features. The Naïve Bayes model was trained with different input vectors (changing only the *k*-mer content feature) and the accuracy was calculated for each model by a tenfold cross validation, dividing the data in 10 subsets, leaving one out and doing the training on the remaining 9. The “left out” dataset is used as test-set.

**Fig. 3:**
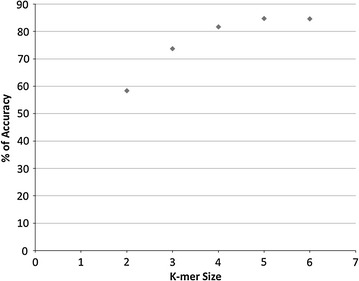
Effect of *k* value on the accuracy of the models for primary sequence

Between *k*-mer sizes from 2 to 5, as the value of *k* increased, the accuracy also increased. After 5-mers, accuracy dropped (Fig. 3). For *k*-mers of length 7 or higher, frequencies could not be evaluated due to the fact that Weka is implemented in Java and it needs significant amounts of memory to run large datasets such as data of vectors with more than 16,000 features. After this analysis, the best features to construct the vector were: *k*-mer size of 5 (1024 possible 5-mers or features), sequence length normalized by the average length of the group and GC content expressed as percentage. This resulted in a vector of 1026 attributes.

#### Training set influence

As shown above, there were some fungal genera that have only one sequence affiliated whereas others were represented by more than 100 sequences (Fig. 2). In order to obtain the best classifier, three datasets were tested to construct the models. Each dataset was composed by a training set for training the algorithm and by a test set used to estimate the accuracy. The three datasets varied in terms of the minimum number of sequences per class (see Table 3; N models: 0–2).

**Table 3:** Accuracy of the model constructed with different training sets at genus level

N model	Dataset (N seq/class)	N records	N classes	Accuracy (%)
NA	ITS1 (1/genus)	37,632	2112	72.73
0	ITS1 (5/genus)	35,363	822	76.90
1	ITS1 (100/genus)	18,941	75	90.58
2	ITS1 (5/sps)	21,083	510	87.01
NA	CBS	8336	256	86.73
NA	Refseq	831	55	81.47

Number of features were 1027

The number of records (sequence vectors) and the number of classes (fungal genera) decreased as the minimum number of sequences per genus increased. Model accuracies were also affected by the different training datasets; when there were more sequences or records by class the accuracy was higher. Furthermore, a reduction of the dataset was performed at the species level, so that species having 4 or less sequences were removed from the dataset. As a result of this filter we ended up having a dataset with less genera, but with more sequences per genera (Table 3; Model 2).

In order to analyse the use of an already curated database versus our own curated database, ITS sequences from CBS and Refseq were downloaded and only full length ITS1 sequences were selected. As done for our own ITS1 database, the genera included for building the classifier were the ones which had a minimum of five sequences. These two databases did not improve the classification power due to a reduction of information (Table 3), as there were less sequences per genus. These results show that our database works as well as a curated external database, with the advantage that our database includes more sequences per genus and the classifier performed with better accuracy.

### Model selection

**Fig. 4:**
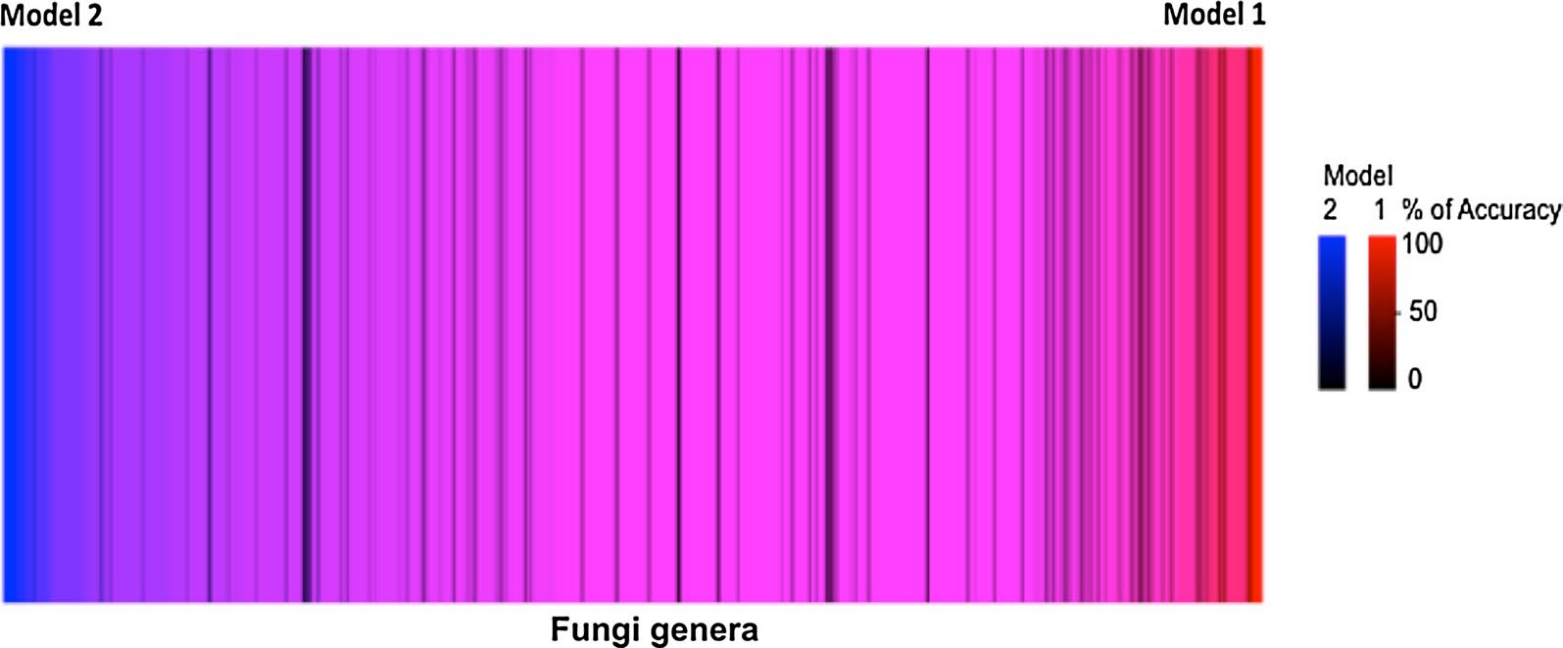
Heat map through the 510 common fungal genera of the selected models 1 and 2. The data (genera in the* X axis*) represented by lines were organized according to model performances. The* left side* shows genera with the best prediction by model 2 (*blue*) and the* right side* indicates those with better accuracies by model 1 (*red*). The intensity of *blue* and *red* colors indicates the percentage of accuracy of the model 2 and 1, respectively

Table 3 shows the accuracy of the N models constructed with different training sets, as we mentioned in the section above. The Naïve Bayes models number 1 and 2 were selected since they covered a greater number of genera and yielded high accuracies. These two models were evaluated to see if the same genera were being correctly classified or if each classifier worked better for particular fungal genera. Figure 4 shows the behaviour of the two classifiers for the 510 genera that were common between the two. Blue represents taxa that were better classified by model 2, red represents taxa that was better classified by model 1. Color intensity represents the accuracy of each model and color overlaps represent taxa for which both models had similar classification accuracy. The center of the graphic (violet region) represents taxa for which the performance of both models was equally high. Overall, the performance of approximately 39 % of the genera was the same in both models. The figure also shows that the predominant color is blue, indicating that model 2 had better performance than model 1 (an additional file shows the background data of the Fig. 4; see Additional file 1: Table S1). Therefore model 2 was selected for the classification.

### Performance of Naïve Bayesian classifiers versus BLASTN

In order to have a control for comparison, classification using model 2 was compared against BLASTN using the same training set as the blast database and the test set as query sequences. The accuracy of the results obtained using the BLASTN approach was 94 %, similar and slightly higher to the obtained using model 2 (87 %).

## Discussion

Accurate sequence classification is a crucial factor to assess fungal community diversity and ecological studies. At present, high-throughput sequencing technologies produce millions of sequences such as ITS1 with no bioinformatics tool to correctly classify such sequences. Supervised machine learning approaches have been very accurate in several bioinformatic prediction methods. This work describes the construction of a fungal ITS1 database based on criteria that would minimize incorrect assignment of taxa, and its use in the development of a machine learning-based classifier.

Besides the fact that reliable reference databases and taxonomies are critical to assign sequence reads to their right phylogenetic affiliation, the development of supervised machine learning classifiers needs curated data for the consequent construction of training and exploration data sets. In the absence of a curated fungal ITS1 database, the first goal was the construction of a curated database of fungal ITS1 sequences. The primary sequence data source (ITS1 sequences from GenBank that recovers Refseq, CBS and nr database sequences) had to be filtered out due to the presence of low-quality sequence data and inaccurate taxonomic information deposited.

The taxonomic and clustering filters were the steps where more sequences were discarded, as shown in Fig. 1. In public databases there are too many fungal ITS1 sequences lacking taxonomic annotation at the genus level and also many environmental sequences without a good annotation [17]. Redundancy was reduced in the clustering step, removing all identical sequences and also the ones that were subsequences of other longer sequences. Nilsson et al. [18] reported that interspecific variability varies throughout the different fungal species and there is not a unifying stringent upper limit for defining all fungal species. In fact, we applied several clustering parameters in order to determine the percentage of identity that would best define natural groups in the fungal kingdom. Our result indicates that there is no sweet spot to determine this, as variation between taxa is as large as variation within taxa (data not shown). In addition to this, clustering even at high percent identity (99 %) led to a dataset with insufficient coverage for many fungal taxa. However, we selected 99 % to avoid loosing taxonomic resolution, while still reducing sequence redundancy.

The reduction of the dataset affected classification accuracy since the number of data per class in learning algorithms is low and can lead to over fitting and suboptimal performances [19]. Indeed, this phenomenon was shown in the classification accuracies of the different models constructed according to the number of sequences allowed for each fungal genus.

Naïve Bayes classifiers have been developed to classify other sequences commonly used for bacterial and fungal barcoding but that cannot be aligned accurately, such as the RDP classifier [16, 20]. The RDP classifier is a Naïve bayes classifier, fast and effective for bacterial and archaeal organisms based on the 16S ribosomal RNA gene. Recently, this classifier has been adapted for the identification of fungal sequences using two markers, the 28S rRNA subunit and the ITS region. For the last one, UNITE and the Warcup ITS training set (sequences retrieved from the UNITE + INSD datasets) are used [21]. Deshpande et al. reported the same problem as we had in classification of fungal ITS sequences due to taxa coverage since several orders could not be represented in the Warcup ITS training set [21].

Mycofier is a classifier that was built to accurately classify fungal ITS1 sequences, choosing the best features based on a curated database of only ITS1 sequences. Indeed Mycofier was developed to specifically classify fungi based on ITS1 sequences.

Although the BLAST approach provided higher classification accuracy, our classifier does not require a pairwise sequence alignment step, which improves speed and lowers computational demands. The accuracy of our classifier is above 87 %. This is an initial effort to develop a machine learning-based classifier for large sequence data sets of hyper variable nature like the ITS1. The ITS1 database and consequently Mycofier are based on the availability of high quality sequences. Development of a classifier with more coverage will be accomplished with the inclusion of underrepresented taxa in the future.

## Conclusions

This study reports the Mycofier tool for the classification of fungal ITS1 sequences. This classifier includes a novel and curated training data set built with a set of sequences from specialized and curated databases. Our training set can still be improved by including good-quality, curated sequence data to improve coverage. Therefore, the classification tool coupled to the use of this database provides accurate identification of ITS1 fungal sequences obtained from NGS technologies. The features used for building the Mycofier classifier make it advantageous over BLAST because it does not require and it is not limited to a pairwise comparison between two sequences. Given its probabilistic nature, Mycofier also captures sequence diversity within the model, this is something that is not available in BLAST searches. In addition, our classifier represents an alternative to phylogenetic placement methods such as pplacer [22].

## Methods

### The ITS1 database

An ITS1 fungal sequence database was constructed by downloading sequences from NCBI GenBank (http://www.ncbi.nlm.nih.gov/; accessed May 9, 2012). A set of 384,542 fungal ITS1 sequences was downloaded using the taxonomic ID 4751 (fungi) and the query word ‘ITS1’. Taxonomic information was obtained from the NCBI taxonomy database by using the BioSQL schema (http://www.biosql.org/) and an in-house Perl script. Approximately 80 % of the total set of GenBank sequences were discarded due to inconsistent taxonomical information. These included sequences lacking binomials, sequences that did not have phylogenetic information at genus level, some *incertae sedis* groups and sequences from environmental samples. An additional 5813 sequences lacking genomic coordinates in their genbank record were also excluded, meaning only full length ITS1 sequences were considered. To reduce redundancy in the data, a clustering algorithm was applied at 100 % identity using UCLUST [23]. The result was 38,939 clusters. The ITS extractor software from UNITE database [24, 25] was used for the identification and extraction of the ITS1 region of the last set of sequences. The last filter was applied to the sequences excluding the ones with less than 100 and more than 400 bases long. A schematic representation of this process is shown in Fig. 1.

### Classifier

#### Training and test data

The sequences of the ITS1 Database were filtered out to obtain only genera that had at least five representative sequences. This set contained the remaining 35,363 sequences, 20 % of them were used to construct the *test set* and 80 % were used to build the *training set* for classification analyses (Fig. 1).

#### Refseq and CBS datasets

Sequences from the Centraalbureau voor Schimmelcultures (CBS) Fungal Biodiversity Centre (http://www.cbs.knaw.nl/) and the new collection of ITS sequences from the Refseq database [26] were used as controls to evaluate the quality of the input data for building the Naïve Bayesian classifier.

#### Feature selection and vector construction

The Weka machine learning workbench [27] was used to build the models based on Naïve Bayes algorithm. Using in-house Perl scripts, the arff files (input for Weka) were built with different feature types and vector sizes. This step was necessary in order to determine the best set of features for the final vector. Vector classes were generated for each genus the sequences belonged to. Features from primary sequences, such as *k*-mer frequency, normalized length (individual sequence length/average length of the entire dataset of sequences) and percent CG were also used as features for the vectors.

In order to select those features in each vector that had more predictive power, the CfSubsetEval and InfoGainAttributeEval feature selection tools implemented in Weka were used. The first one evaluates the worth of a subset of features by considering the individual predictive ability of each feature along with the degree of redundancy between them. The second tool evaluates the worth of a feature by measuring the information gain with respect to the class. These kinds of tools are related to the analysis of variance and are implemented in Weka because of their good performance-selecting features for machine learning algorithms.

### Comparison against BLASTN classification

BLASTN is commonly used to classify rRNA gene sequences and so far it is the only available bioinformatic tool used for ITS1. It was used here for comparison purposes. BLAST+ [28] was downloaded from GenBank (http://www.ncbi.nlm.nih.gov/) and installed locally. The training dataset was used as the database (subjects) and the queries were the sequences of the testing dataset. BLASTN parameters were set to the default values except for an E-value threshold of 0.0001. Best hits from the BLAST search (the ones with lowest E-value and highest bitscore) were parsed out in order to get both their taxonomic information at the genus level and to evaluate accuracy as the percentage of correctly assigned taxa.

## Additional file

10.1186/s13104-016-2203-3 Comparisons of the accuracy percentage obtained by the two models (1 and 2) for each genus.

## Abbreviations

SSU: small subunit
LSU: large-subunit
ITS: internal transcribed spacer
ITS1: internal transcribed spacer region 1
RDP: ribosomal database project
CBS: Centraalbureau voor Schimmelcultures
BLAST: Basic Local Alignment Search Tool
