# Supplementary material for: Mycofier: a new machine learning-based classifier for fungal ITS sequences
Source: BMC Res Notes. 2016 Aug 11;9:402. doi: 10.1186/s13104-016-2203-3 (PMC4982325; doi:10.1186/s13104-016-2203-3)
Supplement: Supplementary file 1 — 10.1186/s13104-016-2203-3 Comparisons of the accuracy percentage obtained by the two models (1 and 2) for each genus. [file 13104_2016_2203_MOESM1_ESM.doc]

|  | **% of accuracy** | |  |  |
| --- | --- | --- | --- | --- |
| **Genus** | **model 1** | **model 2** | **1minus2** | **2minus1** |
| Macowanites | 0 | 100 | -100 | 100 |
| Discula | 0 | 100 | -100 | 100 |
| Evernia | 0 | 100 | -100 | 100 |
| Fusicoccum | 0 | 100 | -100 | 100 |
| Alpova | 0 | 100 | -100 | 100 |
| Pestalotiopsis | 9.433962264 | 100 | -90.56603774 | 90.56603774 |
| Cochliobolus | 12.5 | 100 | -87.5 | 87.5 |
| Paecilomyces | 14.28571429 | 100 | -85.71428571 | 85.71428571 |
| Phialophora | 16.66666667 | 100 | -83.33333333 | 83.33333333 |
| Pleospora | 20 | 100 | -80 | 80 |
| Acremonium | 25 | 100 | -75 | 75 |
| Scytalidium | 25 | 100 | -75 | 75 |
| Mycosphaerella | 15 | 87.5 | -72.5 | 72.5 |
| Protoglossum | 33.33333333 | 100 | -66.66666667 | 66.66666667 |
| Roccellina | 33.33333333 | 100 | -66.66666667 | 66.66666667 |
| Setosphaeria | 33.33333333 | 100 | -66.66666667 | 66.66666667 |
| Lasiosphaeria | 33.33333333 | 100 | -66.66666667 | 66.66666667 |
| Penicillium | 32.84671533 | 98.68421053 | -65.8374952 | 65.8374952 |
| Sphaerulina | 40 | 100 | -60 | 60 |
| Xanthomendoza | 42.85714286 | 100 | -57.14285714 | 57.14285714 |
| Herpotrichia | 50 | 100 | -50 | 50 |
| Camillea | 50 | 100 | -50 | 50 |
| Apiosporina | 50 | 100 | -50 | 50 |
| Amyloporia | 50 | 100 | -50 | 50 |
| Gymnascella | 50 | 100 | -50 | 50 |
| Pyxine | 50 | 100 | -50 | 50 |
| Placopyrenium | 50 | 100 | -50 | 50 |
| Flavopunctelia | 50 | 100 | -50 | 50 |
| Ceratorhiza | 50 | 100 | -50 | 50 |
| Apiognomonia | 50 | 100 | -50 | 50 |
| Didymella | 50 | 100 | -50 | 50 |
| Ophiostoma | 53.19148936 | 100 | -46.80851064 | 46.80851064 |
| Usnea | 53.42465753 | 98.30508475 | -44.88042721 | 44.88042721 |
| Rosellinia | 57.14285714 | 100 | -42.85714286 | 42.85714286 |
| Fomitopsis | 57.14285714 | 100 | -42.85714286 | 42.85714286 |
| Cystoderma | 60 | 100 | -40 | 40 |
| Rhizomucor | 60 | 100 | -40 | 40 |
| Chaetomium | 60 | 100 | -40 | 40 |
| Metacordyceps | 62.5 | 100 | -37.5 | 37.5 |
| Antrodia | 37.5 | 75 | -37.5 | 37.5 |
| Ophiocordyceps | 50 | 87.5 | -37.5 | 37.5 |
| Neonectria | 63.63636364 | 100 | -36.36363636 | 36.36363636 |
| Cytospora | 64.28571429 | 100 | -35.71428571 | 35.71428571 |
| Xanthoria | 44.44444444 | 80 | -35.55555556 | 35.55555556 |
| Emmonsia | 66.66666667 | 100 | -33.33333333 | 33.33333333 |
| Phloeospora | 66.66666667 | 100 | -33.33333333 | 33.33333333 |
| Endothia | 66.66666667 | 100 | -33.33333333 | 33.33333333 |
| Phaeoacremonium | 66.66666667 | 100 | -33.33333333 | 33.33333333 |
| Pseudevernia | 66.66666667 | 100 | -33.33333333 | 33.33333333 |
| Gloeoporus | 66.66666667 | 100 | -33.33333333 | 33.33333333 |
| Moniliophthora | 66.66666667 | 100 | -33.33333333 | 33.33333333 |
| Sporothrix | 66.66666667 | 100 | -33.33333333 | 33.33333333 |
| Lecanora | 66.66666667 | 100 | -33.33333333 | 33.33333333 |
| Heterobasidion | 66.66666667 | 100 | -33.33333333 | 33.33333333 |
| Hortaea | 66.66666667 | 100 | -33.33333333 | 33.33333333 |
| Chalara | 66.66666667 | 100 | -33.33333333 | 33.33333333 |
| Nectria | 50 | 83.33333333 | -33.33333333 | 33.33333333 |
| Cystocoleus | 66.66666667 | 100 | -33.33333333 | 33.33333333 |
| Corynespora | 66.66666667 | 100 | -33.33333333 | 33.33333333 |
| Wilcoxina | 66.66666667 | 100 | -33.33333333 | 33.33333333 |
| Xerula | 66.66666667 | 100 | -33.33333333 | 33.33333333 |
| Dendryphion | 66.66666667 | 100 | -33.33333333 | 33.33333333 |
| Scedosporium | 66.66666667 | 100 | -33.33333333 | 33.33333333 |
| Melanelixia | 66.66666667 | 100 | -33.33333333 | 33.33333333 |
| Microdochium | 66.66666667 | 100 | -33.33333333 | 33.33333333 |
| Battarrea | 66.66666667 | 100 | -33.33333333 | 33.33333333 |
| Aureobasidium | 68.42105263 | 100 | -31.57894737 | 31.57894737 |
| Phomopsis | 70 | 100 | -30 | 30 |
| Paraconiothyrium | 71.42857143 | 100 | -28.57142857 | 28.57142857 |
| Oidium | 71.42857143 | 100 | -28.57142857 | 28.57142857 |
| Aspicilia | 71.42857143 | 100 | -28.57142857 | 28.57142857 |
| Lepiota | 71.42857143 | 100 | -28.57142857 | 28.57142857 |
| Gymnopilus | 72.72727273 | 100 | -27.27272727 | 27.27272727 |
| Phialocephala | 73.33333333 | 100 | -26.66666667 | 26.66666667 |
| Malassezia | 57.14285714 | 83.33333333 | -26.19047619 | 26.19047619 |
| Umbelopsis | 75 | 100 | -25 | 25 |
| Muscodor | 75 | 100 | -25 | 25 |
| Gloeophyllum | 75 | 100 | -25 | 25 |
| Pyrenochaeta | 75 | 100 | -25 | 25 |
| Zasmidium | 75 | 100 | -25 | 25 |
| Geomyces | 75 | 100 | -25 | 25 |
| Eutypella | 75 | 100 | -25 | 25 |
| Sporodictyon | 75 | 100 | -25 | 25 |
| Fusicladium | 25 | 50 | -25 | 25 |
| Peniophora | 75 | 100 | -25 | 25 |
| Polycauliona | 75 | 100 | -25 | 25 |
| Eudarluca | 75 | 100 | -25 | 25 |
| Microsporum | 75 | 100 | -25 | 25 |
| Collophora | 75 | 100 | -25 | 25 |
| Sebacina | 75 | 100 | -25 | 25 |
| Alternaria | 72.09302326 | 94.91525424 | -22.82223098 | 22.82223098 |
| Cercospora | 64.51612903 | 86.95652174 | -22.44039271 | 22.44039271 |
| Bryoria | 77.77777778 | 100 | -22.22222222 | 22.22222222 |
| Annulohypoxylon | 77.77777778 | 100 | -22.22222222 | 22.22222222 |
| Pleurotus | 74.19354839 | 95.65217391 | -21.45862553 | 21.45862553 |
| Sporobolomyces | 78.57142857 | 100 | -21.42857143 | 21.42857143 |
| Botryosphaeria | 78.57142857 | 100 | -21.42857143 | 21.42857143 |
| Puccinia | 76.78571429 | 97.56097561 | -20.77526132 | 20.77526132 |
| Rhizoscyphus | 80 | 100 | -20 | 20 |
| Gyalolechia | 80 | 100 | -20 | 20 |
| Ceratocystiopsis | 80 | 100 | -20 | 20 |
| Hypotrachyna | 80 | 100 | -20 | 20 |
| Schizophyllum | 60 | 80 | -20 | 20 |
| Tolypocladium | 80 | 100 | -20 | 20 |
| Zygosaccharomyces | 80 | 100 | -20 | 20 |
| Pichia | 80 | 100 | -20 | 20 |
| Conocybe | 80 | 100 | -20 | 20 |
| Sporisorium | 80 | 100 | -20 | 20 |
| Rhodocollybia | 80 | 100 | -20 | 20 |
| Leohumicola | 80 | 100 | -20 | 20 |
| Dasyspora | 80 | 100 | -20 | 20 |
| Cladia | 80 | 100 | -20 | 20 |
| Nematoctonus | 80 | 100 | -20 | 20 |
| Clitopilus | 80 | 100 | -20 | 20 |
| Septoria | 47.05882353 | 66.66666667 | -19.60784314 | 19.60784314 |
| Pseudocyphellaria | 81.25 | 100 | -18.75 | 18.75 |
| Morchella | 81.25 | 100 | -18.75 | 18.75 |
| Polyporus | 81.48148148 | 100 | -18.51851852 | 18.51851852 |
| Heterodermia | 81.81818182 | 100 | -18.18181818 | 18.18181818 |
| Verrucaria | 81.81818182 | 100 | -18.18181818 | 18.18181818 |
| Tephromela | 81.81818182 | 100 | -18.18181818 | 18.18181818 |
| Phoma | 5.128205128 | 22.22222222 | -17.09401709 | 17.09401709 |
| Cordyceps | 27.77777778 | 44.82758621 | -17.04980843 | 17.04980843 |
| Hypoderma | 33.33333333 | 50 | -16.66666667 | 16.66666667 |
| Coprinus | 33.33333333 | 50 | -16.66666667 | 16.66666667 |
| Hydnellum | 83.33333333 | 100 | -16.66666667 | 16.66666667 |
| Biscogniauxia | 83.33333333 | 100 | -16.66666667 | 16.66666667 |
| Auricularia | 50 | 66.66666667 | -16.66666667 | 16.66666667 |
| Tomentella | 83.33333333 | 100 | -16.66666667 | 16.66666667 |
| Botrytis | 83.33333333 | 100 | -16.66666667 | 16.66666667 |
| Pilobolus | 83.33333333 | 100 | -16.66666667 | 16.66666667 |
| Serpula | 84.61538462 | 100 | -15.38461538 | 15.38461538 |
| Curvularia | 84.61538462 | 100 | -15.38461538 | 15.38461538 |
| Lecidea | 84.61538462 | 100 | -15.38461538 | 15.38461538 |
| Isaria | 84.61538462 | 100 | -15.38461538 | 15.38461538 |
| Marasmius | 85 | 100 | -15 | 15 |
| Bipolaris | 60 | 75 | -15 | 15 |
| Exophiala | 79.16666667 | 94.11764706 | -14.95098039 | 14.95098039 |
| Teratosphaeria | 85.29411765 | 100 | -14.70588235 | 14.70588235 |
| Cylindrocladium | 45.45454545 | 60 | -14.54545455 | 14.54545455 |
| Postia | 85.71428571 | 100 | -14.28571429 | 14.28571429 |
| Myrothecium | 85.71428571 | 100 | -14.28571429 | 14.28571429 |
| Mycena | 85.71428571 | 100 | -14.28571429 | 14.28571429 |
| Daldinia | 85.71428571 | 100 | -14.28571429 | 14.28571429 |
| Derxomyces | 85.71428571 | 100 | -14.28571429 | 14.28571429 |
| Rasamsonia | 85.71428571 | 100 | -14.28571429 | 14.28571429 |
| Cadophora | 85.71428571 | 100 | -14.28571429 | 14.28571429 |
| Pseudocercospora | 86.36363636 | 100 | -13.63636364 | 13.63636364 |
| Lyophyllum | 66.66666667 | 80 | -13.33333333 | 13.33333333 |
| Coprinellus | 86.66666667 | 100 | -13.33333333 | 13.33333333 |
| Cortinarius | 75.43859649 | 88 | -12.56140351 | 12.56140351 |
| Scleroderma | 87.5 | 100 | -12.5 | 12.5 |
| Rhizophlyctis | 87.5 | 100 | -12.5 | 12.5 |
| Parmelia | 87.5 | 100 | -12.5 | 12.5 |
| Dactylellina | 87.5 | 100 | -12.5 | 12.5 |
| Hymenoscyphus | 87.5 | 100 | -12.5 | 12.5 |
| Byssochlamys | 87.5 | 100 | -12.5 | 12.5 |
| Athelia | 87.5 | 100 | -12.5 | 12.5 |
| Pleopsidium | 87.5 | 100 | -12.5 | 12.5 |
| Leucoagaricus | 87.5 | 100 | -12.5 | 12.5 |
| Xylaria | 76.66666667 | 88.88888889 | -12.22222222 | 12.22222222 |
| Physcia | 88 | 100 | -12 | 12 |
| Tricholoma | 88.37209302 | 100 | -11.62790698 | 11.62790698 |
| Ampelomyces | 66.66666667 | 77.77777778 | -11.11111111 | 11.11111111 |
| Typhula | 88.88888889 | 100 | -11.11111111 | 11.11111111 |
| Togninia | 88.88888889 | 100 | -11.11111111 | 11.11111111 |
| Chaenotheca | 88.88888889 | 100 | -11.11111111 | 11.11111111 |
| Emericella | 88.88888889 | 100 | -11.11111111 | 11.11111111 |
| Guignardia | 88.88888889 | 100 | -11.11111111 | 11.11111111 |
| Dermocybe | 75 | 85.71428571 | -10.71428571 | 10.71428571 |
| Epichloe | 40 | 50 | -10 | 10 |
| Diaporthe | 77.5 | 86.66666667 | -9.166666667 | 9.166666667 |
| Claviceps | 90.90909091 | 100 | -9.090909091 | 9.090909091 |
| Lentinellus | 90.90909091 | 100 | -9.090909091 | 9.090909091 |
| Waitea | 90.90909091 | 100 | -9.090909091 | 9.090909091 |
| Melanoleuca | 90.90909091 | 100 | -9.090909091 | 9.090909091 |
| Phellinus | 83.33333333 | 92.30769231 | -8.974358974 | 8.974358974 |
| Caloplaca | 81.39534884 | 90 | -8.604651163 | 8.604651163 |
| Gymnopus | 91.42857143 | 100 | -8.571428571 | 8.571428571 |
| Ramaria | 92.30769231 | 100 | -7.692307692 | 7.692307692 |
| Coprinopsis | 92.30769231 | 100 | -7.692307692 | 7.692307692 |
| Ganoderma | 92.30769231 | 100 | -7.692307692 | 7.692307692 |
| Arthroderma | 40.625 | 48 | -7.375 | 7.375 |
| Boeremia | 92.85714286 | 100 | -7.142857143 | 7.142857143 |
| Phyllactinia | 92.85714286 | 100 | -7.142857143 | 7.142857143 |
| Coniophora | 92.85714286 | 100 | -7.142857143 | 7.142857143 |
| Trichophyton | 89.74358974 | 96.875 | -7.131410256 | 7.131410256 |
| Hyphoderma | 81.81818182 | 88.88888889 | -7.070707071 | 7.070707071 |
| Cladosporium | 71.42857143 | 78.16091954 | -6.732348112 | 6.732348112 |
| Microbotryum | 93.33333333 | 100 | -6.666666667 | 6.666666667 |
| Hydnum | 60 | 66.66666667 | -6.666666667 | 6.666666667 |
| Wickerhamomyces | 60 | 66.66666667 | -6.666666667 | 6.666666667 |
| Lepraria | 93.33333333 | 100 | -6.666666667 | 6.666666667 |
| Hygrocybe | 93.75 | 100 | -6.25 | 6.25 |
| Hypogymnia | 93.75 | 100 | -6.25 | 6.25 |
| Uromyces | 93.75 | 100 | -6.25 | 6.25 |
| Lasiodiplodia | 94.11764706 | 100 | -5.882352941 | 5.882352941 |
| Clonostachys | 94.11764706 | 100 | -5.882352941 | 5.882352941 |
| Galerina | 94.11764706 | 100 | -5.882352941 | 5.882352941 |
| Candida | 80.32786885 | 86.20689655 | -5.879027699 | 5.879027699 |
| Eutypa | 80 | 85.71428571 | -5.714285714 | 5.714285714 |
| Rhodotorula | 44.44444444 | 50 | -5.555555556 | 5.555555556 |
| Pseudallescheria | 94.44444444 | 100 | -5.555555556 | 5.555555556 |
| Aspergillus | 69.23076923 | 74.7826087 | -5.551839465 | 5.551839465 |
| Hypocrea | 92.42424242 | 97.51552795 | -5.091285526 | 5.091285526 |
| Hypoxylon | 95 | 100 | -5 | 5 |
| Tilletia | 95 | 100 | -5 | 5 |
| Neofusicoccum | 95.45454545 | 100 | -4.545454545 | 4.545454545 |
| Umbilicaria | 95.45454545 | 100 | -4.545454545 | 4.545454545 |
| Lactarius | 95.50561798 | 100 | -4.494382023 | 4.494382023 |
| Xanthoparmelia | 95.89041096 | 100 | -4.109589041 | 4.109589041 |
| Thanatephorus | 33.72093023 | 37.64705882 | -3.926128591 | 3.926128591 |
| Roccella | 96.15384615 | 100 | -3.846153846 | 3.846153846 |
| Mucor | 60.86956522 | 64.70588235 | -3.836317136 | 3.836317136 |
| Verticillium | 90 | 93.75 | -3.75 | 3.75 |
| Pisolithus | 96.77419355 | 100 | -3.225806452 | 3.225806452 |
| Ceratocystis | 93.75 | 96.77419355 | -3.024193548 | 3.024193548 |
| Ilyonectria | 97.05882353 | 100 | -2.941176471 | 2.941176471 |
| Peyronellaea | 93.10344828 | 95.83333333 | -2.729885057 | 2.729885057 |
| Calonectria | 97.2972973 | 100 | -2.702702703 | 2.702702703 |
| Hebeloma | 97.43589744 | 100 | -2.564102564 | 2.564102564 |
| Agaricus | 92.68292683 | 95.23809524 | -2.555168409 | 2.555168409 |
| Rhizophagus | 95.83333333 | 97.91666667 | -2.083333333 | 2.083333333 |
| Inocybe | 96.68874172 | 97.91666667 | -1.227924945 | 1.227924945 |
| Tuber | 98.85057471 | 100 | -1.149425287 | 1.149425287 |
| Hymenopellis | 100 | 100 | 0 | 0 |
| Saccharomyces | 100 | 100 | 0 | 0 |
| Trichothecium | 100 | 100 | 0 | 0 |
| Conidiobolus | 100 | 100 | 0 | 0 |
| Trichosporon | 100 | 100 | 0 | 0 |
| Terriera | 100 | 100 | 0 | 0 |
| Pyrenophora | 100 | 100 | 0 | 0 |
| Terfezia | 100 | 100 | 0 | 0 |
| Ambispora | 100 | 100 | 0 | 0 |
| Erysiphe | 50 | 50 | 0 | 0 |
| Pucciniastrum | 100 | 100 | 0 | 0 |
| Eremothecium | 100 | 100 | 0 | 0 |
| Magnaporthe | 100 | 100 | 0 | 0 |
| Laetiporus | 100 | 100 | 0 | 0 |
| Blumeria | 100 | 100 | 0 | 0 |
| Phlebia | 100 | 100 | 0 | 0 |
| Fomitiporia | 100 | 100 | 0 | 0 |
| Spongipellis | 100 | 100 | 0 | 0 |
| Uwebraunia | 100 | 100 | 0 | 0 |
| Tylospora | 100 | 100 | 0 | 0 |
| Peniophorella | 100 | 100 | 0 | 0 |
| Nigrospora | 100 | 100 | 0 | 0 |
| Tranzschelia | 100 | 100 | 0 | 0 |
| Lewia | 33.33333333 | 33.33333333 | 0 | 0 |
| Oidiodendron | 100 | 100 | 0 | 0 |
| Hamigera | 100 | 100 | 0 | 0 |
| Actinomucor | 100 | 100 | 0 | 0 |
| Golovinomyces | 100 | 100 | 0 | 0 |
| Melanogaster | 100 | 100 | 0 | 0 |
| Stereum | 100 | 100 | 0 | 0 |
| Gliocladium | 100 | 100 | 0 | 0 |
| Cryptosporiopsis | 100 | 100 | 0 | 0 |
| Cetraria | 100 | 100 | 0 | 0 |
| Alectoria | 100 | 100 | 0 | 0 |
| Dioszegia | 100 | 100 | 0 | 0 |
| Flavoparmelia | 100 | 100 | 0 | 0 |
| Diatrype | 50 | 50 | 0 | 0 |
| Phellodon | 100 | 100 | 0 | 0 |
| Thamnolia | 100 | 100 | 0 | 0 |
| Sphaeropsis | 100 | 100 | 0 | 0 |
| Cenococcum | 100 | 100 | 0 | 0 |
| Funneliformis | 100 | 100 | 0 | 0 |
| Pachyphloeus | 100 | 100 | 0 | 0 |
| Monascus | 100 | 100 | 0 | 0 |
| Parastagonospora | 100 | 100 | 0 | 0 |
| Rhizoplaca | 100 | 100 | 0 | 0 |
| Geosmithia | 100 | 100 | 0 | 0 |
| Acarospora | 100 | 100 | 0 | 0 |
| Ramulispora | 0 | 0 | 0 | 0 |
| Allomyces | 100 | 100 | 0 | 0 |
| Gremmeniella | 100 | 100 | 0 | 0 |
| Lophodermium | 100 | 100 | 0 | 0 |
| Asterostroma | 100 | 100 | 0 | 0 |
| Haloguignardia | 100 | 100 | 0 | 0 |
| Punctelia | 100 | 100 | 0 | 0 |
| Cerrena | 100 | 100 | 0 | 0 |
| Collybia | 100 | 100 | 0 | 0 |
| Euoidium | 100 | 100 | 0 | 0 |
| Bjerkandera | 100 | 100 | 0 | 0 |
| Cavernularia | 100 | 100 | 0 | 0 |
| Neocallimastix | 100 | 100 | 0 | 0 |
| Geopora | 100 | 100 | 0 | 0 |
| Dendrographa | 100 | 100 | 0 | 0 |
| Fonsecaea | 100 | 100 | 0 | 0 |
| Dictyonema | 0 | 0 | 0 | 0 |
| Russula | 100 | 100 | 0 | 0 |
| Taiwanofungus | 100 | 100 | 0 | 0 |
| Gaeumannomyces | 100 | 100 | 0 | 0 |
| Melanohalea | 100 | 100 | 0 | 0 |
| Phellopilus | 100 | 100 | 0 | 0 |
| Panellus | 100 | 100 | 0 | 0 |
| Agrocybe | 100 | 100 | 0 | 0 |
| Parmeliopsis | 66.66666667 | 66.66666667 | 0 | 0 |
| Chaetosartorya | 100 | 100 | 0 | 0 |
| Gaertneriomyces | 100 | 100 | 0 | 0 |
| Meliniomyces | 100 | 100 | 0 | 0 |
| Syncephalastrum | 100 | 100 | 0 | 0 |
| Sparassis | 100 | 100 | 0 | 0 |
| Lasiosphaeris | 100 | 100 | 0 | 0 |
| Leotia | 100 | 100 | 0 | 0 |
| Hypocrella | 100 | 100 | 0 | 0 |
| Merimbla | 100 | 100 | 0 | 0 |
| Simplicillium | 100 | 100 | 0 | 0 |
| Therrya | 100 | 100 | 0 | 0 |
| Rhodosporidium | 100 | 100 | 0 | 0 |
| Cystodermella | 100 | 100 | 0 | 0 |
| Liberomyces | 100 | 100 | 0 | 0 |
| Porodaedalea | 100 | 100 | 0 | 0 |
| Stachybotrys | 100 | 100 | 0 | 0 |
| Sydowia | 100 | 100 | 0 | 0 |
| Phanerochaete | 80 | 80 | 0 | 0 |
| Myrmecridium | 100 | 100 | 0 | 0 |
| Diversispora | 100 | 100 | 0 | 0 |
| Acaulospora | 100 | 100 | 0 | 0 |
| Entoleuca | 50 | 50 | 0 | 0 |
| Stereocaulon | 100 | 100 | 0 | 0 |
| Physconia | 100 | 100 | 0 | 0 |
| Purpureocillium | 100 | 100 | 0 | 0 |
| Flavoplaca | 100 | 100 | 0 | 0 |
| Epicoccum | 100 | 100 | 0 | 0 |
| Fellomyces | 100 | 100 | 0 | 0 |
| Calvitimela | 50 | 50 | 0 | 0 |
| Paxillus | 100 | 100 | 0 | 0 |
| Macrophomina | 100 | 100 | 0 | 0 |
| Flavocetraria | 66.66666667 | 66.66666667 | 0 | 0 |
| Thysanophora | 100 | 100 | 0 | 0 |
| Megacollybia | 100 | 100 | 0 | 0 |
| Fuscoporia | 100 | 100 | 0 | 0 |
| Zoophthora | 0 | 0 | 0 | 0 |
| Rhizoctonia | 76.78571429 | 76.78571429 | 0 | 0 |
| Corticium | 0 | 0 | 0 | 0 |
| Kluyveromyces | 100 | 100 | 0 | 0 |
| Nakazawaea | 50 | 50 | 0 | 0 |
| Dermatocarpon | 100 | 100 | 0 | 0 |
| Irpex | 100 | 100 | 0 | 0 |
| Suillus | 100 | 100 | 0 | 0 |
| Tomentellopsis | 100 | 100 | 0 | 0 |
| Biatora | 100 | 100 | 0 | 0 |
| Cladophialophora | 85.71428571 | 85.71428571 | 0 | 0 |
| Phacidiopycnis | 100 | 100 | 0 | 0 |
| Oudemansiella | 66.66666667 | 66.66666667 | 0 | 0 |
| Neofabraea | 100 | 100 | 0 | 0 |
| Amphinema | 100 | 100 | 0 | 0 |
| Plectosphaerella | 100 | 100 | 0 | 0 |
| Pneumocystis | 100 | 100 | 0 | 0 |
| Phellorinia | 50 | 50 | 0 | 0 |
| Saccharata | 100 | 100 | 0 | 0 |
| Nephroma | 100 | 100 | 0 | 0 |
| Imshaugia | 100 | 100 | 0 | 0 |
| Sphaerophorus | 100 | 100 | 0 | 0 |
| Starmerella | 100 | 100 | 0 | 0 |
| Phakopsora | 100 | 100 | 0 | 0 |
| Calvatia | 100 | 100 | 0 | 0 |
| Armillaria | 100 | 100 | 0 | 0 |
| Trapeliopsis | 100 | 100 | 0 | 0 |
| Sawadaea | 100 | 100 | 0 | 0 |
| Choanephora | 100 | 100 | 0 | 0 |
| Grifola | 100 | 100 | 0 | 0 |
| Eurotium | 100 | 100 | 0 | 0 |
| Fomes | 100 | 100 | 0 | 0 |
| Nakaseomyces | 100 | 100 | 0 | 0 |
| Lichtheimia | 100 | 100 | 0 | 0 |
| Parasola | 100 | 100 | 0 | 0 |
| Dufourea | 100 | 100 | 0 | 0 |
| Lachancea | 100 | 100 | 0 | 0 |
| Henrica | 100 | 100 | 0 | 0 |
| Coriolopsis | 100 | 100 | 0 | 0 |
| Ephelis | 100 | 100 | 0 | 0 |
| Paraglomus | 100 | 100 | 0 | 0 |
| Vermispora | 0 | 0 | 0 | 0 |
| Fibroporia | 100 | 100 | 0 | 0 |
| Cyphellophora | 100 | 100 | 0 | 0 |
| Hemileia | 100 | 100 | 0 | 0 |
| Harknessia | 100 | 100 | 0 | 0 |
| Resinicium | 100 | 100 | 0 | 0 |
| Paracoccidioides | 100 | 100 | 0 | 0 |
| Dothiorella | 100 | 100 | 0 | 0 |
| Artomyces | 100 | 100 | 0 | 0 |
| Connopus | 100 | 100 | 0 | 0 |
| Absidia | 100 | 100 | 0 | 0 |
| Claroideoglomus | 100 | 100 | 0 | 0 |
| Ogataea | 100 | 100 | 0 | 0 |
| Cylindrocladiella | 100 | 100 | 0 | 0 |
| Calogaya | 100 | 100 | 0 | 0 |
| Debaryomyces | 100 | 100 | 0 | 0 |
| Phlebiopsis | 50 | 50 | 0 | 0 |
| Hanseniaspora | 100 | 100 | 0 | 0 |
| Hypholoma | 100 | 100 | 0 | 0 |
| Cryphonectria | 88.88888889 | 88.88888889 | 0 | 0 |
| Hypomyces | 100 | 100 | 0 | 0 |
| Dissoconium | 100 | 100 | 0 | 0 |
| Gymnomyces | 100 | 100 | 0 | 0 |
| Alnicola | 100 | 100 | 0 | 0 |
| Marssonina | 100 | 100 | 0 | 0 |
| Cronartium | 100 | 100 | 0 | 0 |
| Ajellomyces | 100 | 100 | 0 | 0 |
| Hyphodermella | 50 | 50 | 0 | 0 |
| Gibellulopsis | 100 | 100 | 0 | 0 |
| Ramalina | 100 | 100 | 0 | 0 |
| Gnomonia | 100 | 100 | 0 | 0 |
| Hypsizygus | 100 | 100 | 0 | 0 |
| Phillipsia | 100 | 100 | 0 | 0 |
| Parmotrema | 100 | 100 | 0 | 0 |
| Strobilurus | 100 | 100 | 0 | 0 |
| Physciella | 100 | 100 | 0 | 0 |
| Filobasidiella | 100 | 100 | 0 | 0 |
| Pleurostomophora | 100 | 100 | 0 | 0 |
| Blakeslea | 50 | 50 | 0 | 0 |
| Peltigera | 100 | 100 | 0 | 0 |
| Sarcinomyces | 100 | 100 | 0 | 0 |
| Batcheloromyces | 100 | 100 | 0 | 0 |
| Xanthopsoroma | 100 | 100 | 0 | 0 |
| Amanita | 95 | 94.44444444 | 0.555555556 | -0.555555556 |
| Talaromyces | 93.10344828 | 92.30769231 | 0.795755968 | -0.795755968 |
| Lentinus | 92.85714286 | 91.66666667 | 1.19047619 | -1.19047619 |
| Cladonia | 98.7804878 | 96.875 | 1.905487805 | -1.905487805 |
| Xerocomus | 92.85714286 | 90.90909091 | 1.948051948 | -1.948051948 |
| Colletotrichum | 38.68613139 | 36.66666667 | 2.01946472 | -2.01946472 |
| Rhizopogon | 90.90909091 | 88.88888889 | 2.02020202 | -2.02020202 |
| Tulasnella | 90 | 87.5 | 2.5 | -2.5 |
| Grosmannia | 46.15384615 | 42.85714286 | 3.296703297 | -3.296703297 |
| Glomerella | 98.30508475 | 94.82758621 | 3.477498539 | -3.477498539 |
| Inonotus | 85.71428571 | 81.81818182 | 3.896103896 | -3.896103896 |
| Leveillula | 91.66666667 | 87.5 | 4.166666667 | -4.166666667 |
| Fusarium | 63.90243902 | 59.67741935 | 4.22501967 | -4.22501967 |
| Diplodia | 57.14285714 | 52.63157895 | 4.511278196 | -4.511278196 |
| Rusavskia | 80 | 75 | 5 | -5 |
| Clavulina | 80 | 75 | 5 | -5 |
| Podosphaera | 100 | 94.73684211 | 5.263157895 | -5.263157895 |
| Leptosphaeria | 88.88888889 | 83.33333333 | 5.555555556 | -5.555555556 |
| Beauveria | 95.45454545 | 89.74358974 | 5.710955711 | -5.710955711 |
| Botryotinia | 40 | 33.33333333 | 6.666666667 | -6.666666667 |
| Trametes | 84.61538462 | 77.27272727 | 7.342657343 | -7.342657343 |
| Mycocalicium | 75 | 66.66666667 | 8.333333333 | -8.333333333 |
| Phaeophyscia | 91.66666667 | 83.33333333 | 8.333333333 | -8.333333333 |
| Glomus | 68.75 | 60 | 8.75 | -8.75 |
| Sclerotinia | 100 | 90 | 10 | -10 |
| Meyerozyma | 90 | 80 | 10 | -10 |
| Coccomyces | 60 | 50 | 10 | -10 |
| Chrysoporthe | 100 | 89.47368421 | 10.52631579 | -10.52631579 |
| Mycoblastus | 100 | 88.88888889 | 11.11111111 | -11.11111111 |
| Stagonosporopsis | 77.77777778 | 66.66666667 | 11.11111111 | -11.11111111 |
| Ceratobasidium | 100 | 87.5 | 12.5 | -12.5 |
| Neosartorya | 100 | 87.5 | 12.5 | -12.5 |
| Laccaria | 100 | 87.5 | 12.5 | -12.5 |
| Venturia | 100 | 87.5 | 12.5 | -12.5 |
| Cetrelia | 80 | 66.66666667 | 13.33333333 | -13.33333333 |
| Anaptychia | 80 | 66.66666667 | 13.33333333 | -13.33333333 |
| Boletus | 90.90909091 | 76.92307692 | 13.98601399 | -13.98601399 |
| Flammulina | 100 | 85.71428571 | 14.28571429 | -14.28571429 |
| Astraeus | 100 | 85.71428571 | 14.28571429 | -14.28571429 |
| Akanthomyces | 66.66666667 | 50 | 16.66666667 | -16.66666667 |
| Pseudoplagiostoma | 66.66666667 | 50 | 16.66666667 | -16.66666667 |
| Leptographium | 100 | 83.33333333 | 16.66666667 | -16.66666667 |
| Drechslera | 66.66666667 | 50 | 16.66666667 | -16.66666667 |
| Hymenogaster | 50 | 33.33333333 | 16.66666667 | -16.66666667 |
| Nemania | 50 | 33.33333333 | 16.66666667 | -16.66666667 |
| Lecanicillium | 88.88888889 | 71.42857143 | 17.46031746 | -17.46031746 |
| Rhizopus | 45.45454545 | 27.5 | 17.95454545 | -17.95454545 |
| Cryptococcus | 85.71428571 | 66.66666667 | 19.04761905 | -19.04761905 |
| Valsa | 75.86206897 | 56.52173913 | 19.34032984 | -19.34032984 |
| Leucophleps | 100 | 80 | 20 | -20 |
| Lobaria | 100 | 80 | 20 | -20 |
| Lentinula | 100 | 80 | 20 | -20 |
| Sarocladium | 77.77777778 | 57.14285714 | 20.63492063 | -20.63492063 |
| Hericium | 100 | 75 | 25 | -25 |
| Monilinia | 100 | 75 | 25 | -25 |
| Psilocybe | 75 | 50 | 25 | -25 |
| Chaetosphaeria | 75 | 50 | 25 | -25 |
| Hirsutella | 100 | 75 | 25 | -25 |
| Neoscytalidium | 100 | 75 | 25 | -25 |
| Auxarthron | 100 | 66.66666667 | 33.33333333 | -33.33333333 |
| Thelephora | 100 | 66.66666667 | 33.33333333 | -33.33333333 |
| Lipomyces | 100 | 66.66666667 | 33.33333333 | -33.33333333 |
| Stemphylium | 100 | 66.66666667 | 33.33333333 | -33.33333333 |
| Phymatotrichopsis | 100 | 66.66666667 | 33.33333333 | -33.33333333 |
| Mucidula | 100 | 66.66666667 | 33.33333333 | -33.33333333 |
| Cunninghamella | 100 | 66.66666667 | 33.33333333 | -33.33333333 |
| Amylomyces | 66.66666667 | 33.33333333 | 33.33333333 | -33.33333333 |
| Microthia | 66.66666667 | 33.33333333 | 33.33333333 | -33.33333333 |
| Protoblastenia | 85.71428571 | 50 | 35.71428571 | -35.71428571 |
| Arthrobotrys | 85.71428571 | 50 | 35.71428571 | -35.71428571 |
| Paraphoma | 75 | 33.33333333 | 41.66666667 | -41.66666667 |
| Pycnoporus | 100 | 50 | 50 | -50 |
| Peziza | 92.85714286 | 42.85714286 | 50 | -50 |
| Otidea | 100 | 50 | 50 | -50 |
| Ulocladium | 100 | 50 | 50 | -50 |
| Podaxis | 50 | 0 | 50 | -50 |
| Melampsora | 100 | 50 | 50 | -50 |
| Orbilia | 50 | 0 | 50 | -50 |
| Coniosporium | 100 | 50 | 50 | -50 |
| Piloderma | 100 | 50 | 50 | -50 |
| Cookeina | 100 | 50 | 50 | -50 |
| Amandinea | 100 | 50 | 50 | -50 |
| Coccocarpia | 100 | 50 | 50 | -50 |
| Hyperphyscia | 100 | 50 | 50 | -50 |
| Neoerysiphe | 100 | 50 | 50 | -50 |
| Tremella | 100 | 50 | 50 | -50 |
| Metarhizium | 88 | 37.5 | 50.5 | -50.5 |
| Trichaptum | 66.66666667 | 0 | 66.66666667 | -66.66666667 |
| Hannaella | 66.66666667 | 0 | 66.66666667 | -66.66666667 |
| Volvariella | 100 | 0 | 100 | -100 |
| Capronia | 100 | 0 | 100 | -100 |
| Rinodina | 100 | 0 | 100 | -100 |
| Scheffersomyces | 100 | 0 | 100 | -100 |
